# Motion Polytopes in Virtual Reality for Shared Control in Remote Manipulation Applications

**DOI:** 10.3389/frobt.2021.730433

**Published:** 2021-09-09

**Authors:** Mark Zolotas, Murphy Wonsick, Philip Long, Taşkın Padır

**Affiliations:** ^1^ Northeastern University, Boston, MA, United States; ^2^ Irish Manufacturing Research, Dublin, Ireland

**Keywords:** human-in-the-loop teleoperation, motion polytopes, shared control, virtual fixtures, virtual reality

## Abstract

In remote applications that mandate human supervision, shared control can prove vital by establishing a harmonious balance between the high-level cognition of a user and the low-level autonomy of a robot. Though in practice, achieving this balance is a challenging endeavor that largely depends on whether the operator effectively interprets the underlying shared control. Inspired by recent works on using immersive technologies to expose the internal shared control, we develop a virtual reality system to visually guide human-in-the-loop manipulation. Our implementation of shared control teleoperation employs end effector manipulability polytopes, which are geometrical constructs that embed joint limit and environmental constraints. These constructs capture a holistic view of the constrained manipulator’s motion and can thus be visually represented as feedback for users on their operable space of movement. To assess the efficacy of our proposed approach, we consider a teleoperation task where users manipulate a screwdriver attached to a robotic arm’s end effector. A pilot study with prospective operators is first conducted to discern which graphical cues and virtual reality setup are most preferable. Feedback from this study informs the final design of our virtual reality system, which is subsequently evaluated in the actual screwdriver teleoperation experiment. Our experimental findings support the utility of using polytopes for shared control teleoperation, but hint at the need for longer-term studies to garner their full benefits as virtual guides.

## 1 Introduction

Teleoperation is a well-established robot control method that plays a pivotal role in complex and unpredictable settings where human supervision is necessary. For instance, remote teleoperation is highly desirable in extreme scenarios where the co-presence of a human operator poses unwanted risk, such as tasks performed underground, underwater, or even in space. However, the direct teleoperation of robotic systems is challenging and often places significant cognitive burden on the operator (Tanwani and Calinon, 2017; Xi et al., 2019; Hetrick et al., 2020). This is especially true when handling robots with high degrees-of-freedom, like robotic arms for grasping and manipulation (Losey et al., 2020). In order to alleviate any excess workload exerted on a teleoperator, shared control is typically employed as a means of providing autonomous assistance.

The shared control paradigm is widely applied in any task where a human operator and robot collaborate towards a common goal by *simultaneously* issuing control over a system (Abbink et al., 2018). Many prior works have demonstrated that by engaging in shared control, a human user can apply their expertise and high-level cognition to the teleoperation task, as well as exploit the precision and accuracy of robot autonomy (Dragan and Srinivasa, 2013; Javdani et al., 2015; Jain and Argall, 2019). Despite these benefits, users may find the arbitration process of shared control a frustrating and bewildering experience. A predominant reason for this is the misalignment between a user’s intended control policy and the actual robot behavior (Zolotas and Demiris, 2019). Even when the robot either correctly infers a human’s goal or knows it *a priori*, the user may not accept the resulting behavior unless administered reassurance through feedback (Dragan and Srinivasa, 2013).

In many instances of shared control, the haptic channel is the selected modality of sensory feedback during this exchange (Abbink et al., 2018; Losey et al., 2018). Indeed, haptic shared control offers numerous benefits, ranging from operator training (O’Malley et al., 2005) to improving teleoperation performance (Selvaggio et al., 2018). Force feedback has also enjoyed success in learning from demonstration applications within shared control (Kucukyilmaz and Demiris, 2018; Zeestraten et al., 2018). Nevertheless, a potential drawback in this medium resides in the limited sensorimotor teaching derived from force feedback. For complex manipulation tasks that necessitate heightened user attention and precision, haptic interfaces by themselves may not provide a sufficiently rich channel to relay information back to users (Losey et al., 2018).

Alternatively, mixed reality headsets are an emerging technology that have recently gained traction in addressing misalignment in shared control (Zolotas and Demiris, 2019; Brooks and Szafir, 2020). These headsets are not without flaws, but in the scope of shared control where users must maintain attention and *actively* participate in the task-at-hand, they hold great promise over other technologies (Sibirtseva et al., 2018). Furthermore, they can supply feedback across multiple modalities, *e.g.,* through visualizations, sounds and haptic vibrations. While these headsets are a suitable medium for exposing the otherwise “black-box” nature of shared control, certain design considerations are crucial to avoid hindering task performance (Zolotas et al., 2018). In particular, the user interface should clearly delineate the range of constraints associated with the remote robot manipulator, including joint limits, proximity to obstacles and singularities. A naive presentation of this information would lead to the teleoperator considering dozens of independent variables at once, risking information overload (Mortimer et al., 2017).

Taking these design considerations into account, we propose a shared control methodology involving on-the-fly generation of *virtual fixtures* for remote teleoperation in virtual reality (VR). Virtual fixtures (Rosenberg, 1992; Bowyer et al., 2014) aim to reduce the cognitive burden on the operator by creating enclosed volumetric zones, inside of which the robot can operate. These zones can be created in numerous ways, *e.g.,* directly from 3D point cloud data (Yamamoto et al., 2012), using shape primitives (Bettini et al., 2004), or manually (Quintero et al., 2017). Although virtual fixtures are typically concerned with the surrounding environment, an optimal system should also incorporate the robot’s intrinsic constraints, such as joint limits and performance capacities. In Long et al. (2019), constraints originating from the environment and robot are embedded into geometrical objects, namely polytopes, to generate a series of virtual guides for guarded teleoperation.

Inspired by the prospects of using polytope-generated virtual guides for *both* assistance and feedback, we develop a polytope-based method of shared control. To establish a tightly coupled relationship between polytopes and shared control, we introduce a novel “shrinking” modification over traditional motion polytopes that arbitrates operator inputs in accordance with their estimated intentions. Unlike most virtual fixture methods of shared control relying on haptic guidance to enhance telepresence (O’Malley et al., 2005; Selvaggio et al., 2018; Zeestraten et al., 2018), ours instead adopts a visually immersive experience through VR. Moreover, most teleoperation works in VR present a robot embodied view of the remote environment (Xi et al., 2019; Hetrick et al., 2020; Wonsick and Padir, 2020). However, recent works have also shown the utility of model-based perspectives (Van de Merwe et al., 2019; Wonsick et al., 2021a). Ergo, we additionally explore the potential benefits of a model-based perspective.

**FIGURE 1:**
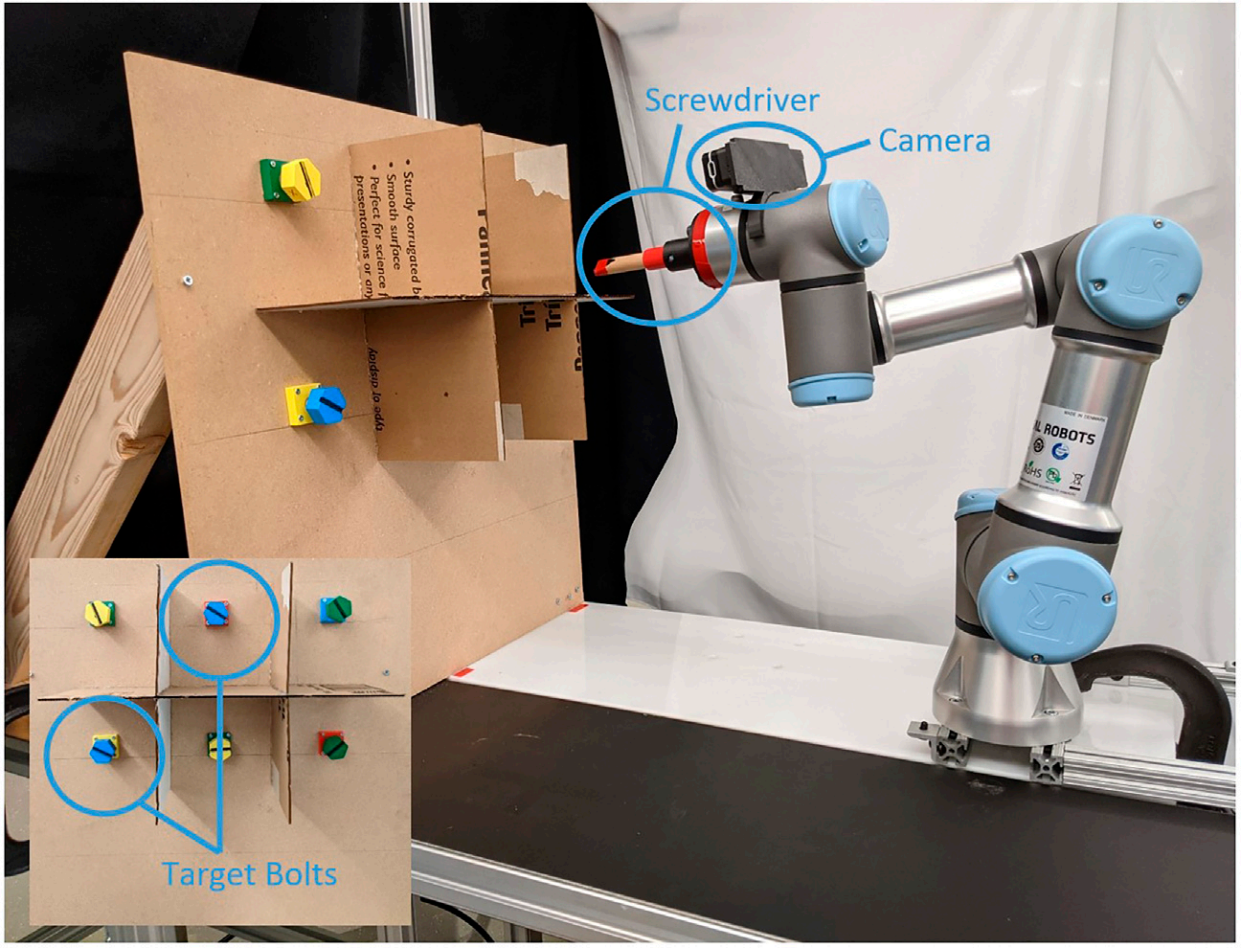
Illustration of the UR3e robotic arm in its initial configuration for the screwdriver experiment. In the bottom left is a front-facing view of the blue target bolts that users were requested to tighten in the teleoperation experiment. The robot’s end effector has been extended to include a wooden screwdriver, as well as a camera for the precision necessary to complete this task.

We investigate the efficacy of our approach by conducting a human-robot interaction experiment where subjects teleoperate a robotic arm in VR to screw in a set of bolts (shown in Figure 1). The task is motivated by the potential advantages of deploying robots to utilize tools under extreme conditions, *e.g.,* nuclear decommissioning (Wonsick et al., 2021b). Prior to this experiment, a pilot user study was also held to designate the most suitable VR interface for the task. Therefore, the main contributions of this paper are: 1) a shared control teleoperation method that relies on virtual fixtures obtained from joint space polytopes to inform the arbitration; 2) a user-informed VR interface for teleoperation with the internal properties of polytopes visualized to graphically aid operators; and 3) a real-world experiment that evaluates how effective the proposed system is when remotely controlling a robotic arm to wield a screwdriver.

The remainder of the paper is organized as follows. Section 2 describes our shared control system, fusing a simple intention estimation algorithm with polytope-guided teleoperation assistance. Our VR system and an array of visualizations are then detailed in Section 3, alongside a pilot user study that helped determine the final interface. In Section 4, we present the remote-operated screwdriver experiment and its results. Sections 5 and 6 reflect on the key insights drawn from our teleoperation experiment and discuss future avenues for research. Closing remarks are then provided in Section 7.

## 2 Shared Control Methodology

In this section, we introduce a shared control methodology tailored to the target domain of teleoperation. Our method falls under the popular scheme of “predict-then-blend” (Javdani et al., 2015), which consists of two core processes: intention estimation and arbitration. A typical interaction cycle of these processes will have the robot first recognize a user’s intentions, *e.g.,* their intended joint configuration, from a set of task-specific goals. The robot will then compute commands that best align with this estimation of intent, and subsequently arbitrate between these autonomous commands and any user inputs to finalize on an optimal outcome for the task objective.

Before proceeding with the implementation of these core mechanisms, we briefly outline in Section 2.1 the chosen manipulator model. Sections 2.2 and 2.3 then describe the intention estimation and arbitration steps, respectively, with the latter employing joint space polytopes for assistive teleoperation. Of the virtual fixture methods previously applied to arbitration (O’Malley et al., 2005; Selvaggio et al., 2018; Zeestraten et al., 2018), we believe this manuscript is the first to depict a use-case for motion polytopes.

### 2.1 Manipulator Model

We consider a manipulator with *n* degrees-of-freedom operating in 6-dimensional space. The end effector pose is represented by a vector $x_{n}\in R^{6}$, consisting of the position $x_{n}^{p}\in R^{3}$ and unit quaternion orientation $x_{n}^{q}\in R^{3}$, all of which can be derived from the forward kinematic chain *fk*:

$$x_{n}=fk\left(q\right),$$ (1)

where **q** = [*q*_1_, … , *q*_*n*_] are the joint configuration variables. The twist at the end effector frame ***ν***_*n*_ can then be obtained from the differential kinematic model:

$$\nu_{n}=\left[\begin{array}{c} v\\ \omega \end{array}\right]=J_{n}\dot{q},$$ (2)

with **v** and ***ω*** denoting translational and angular velocities, respectively. **J**_*n*_ is the 6 × *n* Jacobian matrix defined at the end effector frame *n*, and $\dot{q}={\left[{\dot{q}}_{1},\ldots ,{\dot{q}}_{n}\right]}^{T}$ is the joint velocity vector.

### 2.2 Human Intention Estimation

Estimating a human’s intention is a multidisciplinary subject that plays an integral part in shared control, as it can guide the robot’s decision-making on how to best assist (Demiris, 2007; Jain and Argall, 2019). Throughout this paper, we adopt a loose terminology for *intention information* (Losey et al., 2018), where goals, targets and intentions will be used interchangeably. Furthermore, we assume a discrete goal space G exists for the specific task and that it is known to both the human and robot at run time. For our target domain of assisted teleoperation in screwing bolts, the goal space spans all bolt locations with each possessing an ideal joint state based on pre-recorded expert trajectories. In other words, we store a dictionary of mappings from goal labels to idealistic joint configurations. Hence, the overall aim of our intention estimation process is to infer a user’s goal of interest $\hat{g}\in G$ and yield its corresponding joint configuration $\hat{q}$.

Following an extensive body of literature that uses Hidden Markov Models (HMMs) for human intention estimation (Tanwani and Calinon, 2017; Jain and Argall, 2019; Brooks and Szafir, 2020), we also develop an HMM to infer target bolts from manipulator data. First, we consider a sequence of observations **o**_1:*t*_ = (**o**_1_, … , **o**_*t*_) as the Euclidean distances from the current end effector’s position $x_{t}^{p}$ to all other goals:

$$o_{t}=\|\;x_{t}^{p}-g_{i}^{p}\;\|\;,\forall g_{i}\in G,$$ (3)

where the frame *n* subscript is removed for notational simplicity and $g_{i}^{p}$ are the three position variables of the end effector at the *i* ^th^ bolt. Proximity to goal is a traditional measure of intent in shared control (Dragan and Srinivasa, 2013; Javdani et al., 2015; Jain and Argall, 2019) and serves as the single observation source for our simple task domain.

In the same vein as prior work, we then let the hidden state of the HMM represent the user’s current goal *g*_*t*_ and treat intention estimation as a Bayesian filtering problem where the aim is to derive the posterior probability *P* ( *g*_*t*_ |**o**_1:*t*_) (Jain and Argall, 2019). Many shared control frameworks apply such Bayesian reasoning to maintain a *belief* across all task goals, as the robot can then reason over the entire goal space G to select actions that sustain assistance even under uncertainty (Javdani et al., 2015). Confidence levels surrounding this predictive distribution can also be exploited to dictate how robot-user control is arbitrated.

The posterior probability for a particular goal at time *t*, also known as the belief state *b*_*t*_ (*g*_*t*_), is computed using Bayes rule:

$$b_{t}\left(g_{t}\right)=P\left(g_{t}\;|\;o_{1:t}\right)=\frac{P\left(o_{t}\;|\;g_{t}\right)P\left(g_{t}\;|\;o_{1:t-1}\right)}{P\left(o_{t}\;|\;o_{1:t-1}\right)}\propto P\left(o_{t}\;|\;g_{t}\right)P\left(g_{t}\;|\;o_{1:t-1}\right),$$ (4)

which is simplified in HMMs by the assumption that observations **o**_1:*t*_ exhibit the Markov property of conditional independence across timesteps. Partitioning *P* (*g*_*t*_ |**o**_1:*t*−1_) leads to a recursive update of the belief state:

$$\begin{array}{ll} b_{t}\left(g_{t}\right)=P\left(g_{t}\;|\;o_{1:t}\right) & \propto P\left(o_{t}\;|\;g_{t}\right)\underset{g_{t-1}\in G}{\sum }P\left(g_{t-1}\;|\;o_{1:t-1}\right)P\left(g_{t}\;|\;g_{t-1}\right) \end{array}$$ (5)

$$\begin{array}{ll} & \propto P\left(o_{t}\;|\;g_{t}\right)\underset{g_{t-1}\in G}{\sum }b_{t-1}\left(g_{t-1}\right)P\left(g_{t}\;|\;g_{t-1}\right). \end{array}$$ (6)

where *P* (**o**_*t*_ | *g*_*t*_) and *P* (*g*_*t*_ | *g*_*t*−1_) are the emission and transition probabilities, respectively. As a result, the filtering process boils down to a repeated assignment of posterior probabilities for each g∈G given incoming observations. To complete the HMM description, we configure the starting probability *P* (*g*_0_) to be uniform.

Lastly, the inferred goal ${\hat{g}}_{t}$ is decoded using *maximum a posteriori* estimation as in Jain and Argall (2019):

$${\hat{g}}_{t}={\mathrm{arg max}}_{g_{t}\in G}P\left(g_{t}\;|\;o_{t}\right).$$ (7)

A lookup of ${\hat{g}}_{t}$ in the aforementioned dictionary of goal states to joint configurations will produce the associated ${\hat{q}}_{t}$.

### 2.3 Polytopes for Arbitration

For arbitration in the shared control, we propose to utilize polytopes as a means of generating virtual fixtures during teleoperation. While virtual fixtures often relate to the surrounding environment, optimum performance requires the robot’s intrinsic constraints, such as joint limits, to also be considered. In robotics, the most commonly used performance measure is the manipulability ellipsoid first presented in Yoshikawa (1984). Much work has focused on embedding supplementary information within this ellipsoid such that it considers the effects of joint position constraints (Tsai, 1986), joints velocity limits (Lee, 1997), or obstacles in the environment (Vahrenkamp et al., 2012; Vahrenkamp and Asfour, 2015). In contrast to the manipulability ellipsoid, polytopes give an exact representation of velocity limits (Kokkinis and Paden, 1989) and are less susceptible to error (Krut and Pierrot, 2004). Indeed, as polytopes are geometric objects, they can be combined through union or intersection to represent the capacities of composite serial or parallel chains (Long and Padir, 2020). Finally, since polytopes are represented as a system of linear inequalities, supplementary constraints can be easily added, for example friction cones (Caron et al., 2017), workspace danger zones (Long and Padır, 2018) or a zero-moment point (Rasheed et al., 2018).

We build on the work first proposed in Long et al. (2019), where a *constrained motion polytope* is constructed and used for teleoperation by generating a series of virtual guides. These guides consider the distance-to-collision with environmental obstacles, joint position limits and joint velocity limits. The polytopes present the operator with a Cartesian representation of both the workspace and configuration space constraints during teleoperation in a cluttered environment.

A polytope, P can be represented in two ways: as the convex hull of its vertex set, known as the V-representation and denoted as $P^{V}$, or as volume bounded by a finite number of half-spaces, known as the H-representation and denoted as $P^{H}$, written respectively as:

$$P^{V}=\left\{x:x=\overset{n}{\underset{i=1}{\sum }}\alpha_{i}y_{i}\left|\right.|\alpha_{i}\geq 0,\overset{n}{\underset{i=1}{\sum }}\alpha_{i}=1\right\},\;P^{H}=Ax\leq b,$$ (8)

where **y**_*i*_ denotes the *i*th element of the vertex set and **x** is any point inside P, **A** contains the half-spaces’ normals, and **b** is the shifted distance from the origin along the normal. Converting between the V and H representations can be carried out in several ways, for example using the double description method (Fukuda and Prodon, 1996). The manipulability of a serial manipulator can be obtained by first constructing the joint space polytope in H-representation:

$$Q^{H}=\left[\begin{array}{c} I_{n}\\ -I_{n} \end{array}\right]\dot{q}\leq \left[\begin{array}{c} {\dot{q}}_{max}\\ -{\dot{q}}_{min} \end{array}\right],$$ (9)

where **I**_*n*_ is the *n* × *n* identity matrix and ${\dot{q}}_{max}$ and ${\dot{q}}_{min}$ are robot’s maximum and minimum joint velocities. Using the double description method, an equivalent polytope defined by its vertices is written as:

$$Q^{V}=\left\{\begin{array}{ccc} {\dot{q}}_{1}^{v}, & {\dot{q}}_{2}^{v}, & \ldots ,{\dot{q}}_{2^{n}}^{v} \end{array}\right\},$$ (10)

where ${\dot{q}}_{i}^{v}$ denotes the *i*th vertex of Q. A manipulability polytope, denoted as P, representing the Cartesian-space velocities can then be obtained by transforming the vertices of (Eq. 10) to Cartesian space using (Eq. 2). P’s vertex set representation is given as:

$$P^{V}=\left\{\begin{array}{cc} \nu_{1}^{v} & \ldots \nu_{2^{n}}^{v} \end{array}\right\}=\left\{\begin{array}{cc} J_{n}{\dot{q}}_{1}^{v}\ldots & J_{n}{\dot{q}}_{2^{n}}^{v} \end{array}\right\}.$$ (11)

The convexity of a polytope is preserved under affine transformation, thus a bounded volume of P can be easily obtained which represents the system’s manipulability and serves as an exact indicator of robot performance.

To obtain the *constrained motion polytope* for a manipulator, a joint space polytope is first constructed using position deviations instead of instantaneous velocities.

In order to reformulate (Eq. 11) for allowable motions, the robot’s motions are discretized into timesteps. At timestep *k*, let the robot’s pose be denoted as **x**_*k*_ and the instantaneous joint velocity as ${\dot{q}}_{k}$. Over a period of *δt* seconds, assume the end effector travels a distance of *δ* **x**_*k*_, then the resulting end-effector pose at instant *k* + 1 can be obtained by linearizing (Eq. 1) using (Eq. 2), namely:

$$\begin{array}{ll} x_{k+1} & =x_{k}+\delta x_{k}\approx fk\left(q_{k}+\delta q_{k}\right), \end{array}$$ (12)

$$\begin{array}{ll} \delta x_{k} & =J_{n}\delta q_{k} \end{array}$$ (13)

where *δ* **q**_*k*_ denotes the displacement of the joint variables over the timestep and is defined as:

$$\;\delta q_{k}={\dot{q}}_{k}\;\delta t.$$ (14)

For a point I on the robot’s kinematic chain whose position with respect to the world frame is denoted by the vector **r**_*i*_, the displacement due to *δ* **q**_*k*_ is written as:

$$\delta x_{k}^{i}=J_{i}\delta q_{k},$$ (15)

where $J_{i}\in R^{3\times n}$ denotes the kinematic Jacobian matrix that relates the velocities of the preceding joints in kinematic chain to the translational velocity at point I. Using the above linearization, limits of translational motion for any point on the manipulator body can be defined based on the location of environmental obstacles. The translational motion of point I *towards* an environmental obstacle O, denoted as $\delta x_{o,k}^{i}$, is defined as:

$$\delta x_{o,k}^{i}={\hat{r}}_{io}^{T}\;J_{i}\;\delta q_{k},$$ (16)

where **r**_*io*_ = **r**_*i*_ −**r**_*o*_ is the relative position between O and I, ‖**r**_*io*_‖ is the norm of this vector, *i.e.,* the distance, while ${\hat{r}}_{io}$ denotes the corresponding normalized unit vector. Hence, to prevent a potential collision between I and O, an allowable motion constraint defining the maximum displacement of I can be expressed as follows:

$${\hat{r}}_{io}^{T}\;J_{i}\;\delta q_{k}\leq \|r_{io}\|,$$ (17)

This can be repeated for a set of *l* points discretized along the kinematic chain, leading to the following set of linear inequalities:

$$[\begin{array}{c} {\hat{r}}_{1o}^{T}\;J_{1}\\ \vdots \\ {\hat{r}}_{lo}^{T}\;J_{l} \end{array}]\delta q_{k}\leq [\begin{array}{c} \left\|r_{1o}\right\|\\ \vdots \\ \left\|r_{lo}\right\| \end{array}].$$ (18)

In our implementation, we select at every instant the point along each link nearest to an environmental obstacle, *i.e.,* *l* = *n*. Hence (Eq. 18) represents the set of instantaneous collision-free joint deviations for each link on the robot’s kinematic chain, which is then repeated for all obstacles in the environment.

Aside from obstacles, a robot’s motion is restricted by positional limits of the joints. To integrate these into the polytope, the following linear inequalities are included:

$$\left[\begin{array}{c} I_{n}\\ -I_{n} \end{array}\right]\delta q_{k}\leq \left[\begin{array}{c} q_{max}-q_{k}\\ q_{k}-q_{min} \end{array}\right],$$ (19)

where **q**_*max*_ and **q**_*min*_ are vectors of upper and lower positional joint limits, respectively.

Finally, it should be noted that the linearization error increases as the joint displacement increases, thus a maximum limit is imposed to ensure a satisfactory approximation of link motion:

$$\left[\begin{array}{c} I_{n}\\ -I_{n} \end{array}\right]\delta q_{k}\leq \left[\begin{array}{c} \delta q_{max}\\ \delta q_{max} \end{array}\right],$$ (20)

where *δ* **q**_*max*_ is the vector of linearization limits for each joint of the kinematic chain. Increasing the values of *δ* **q**_*max*_ expands the free space polytope at a cost of reduced fidelity. While not necessary, for simplicity, we let *δq*_1_ = *δq*_2_ = … = *δq*_*n*_ = *ϵ*_*lin*_. The linearization limits can, for various reasons, be altered at run time. For instance, limits could be increased to enlarge the solution space in (Eq. 22). Alternatively, the limits could be shrunk progressively in order to guide the user towards a defined configuration as shown in (Eq. 24).

By stacking (Eqs. 18–20), an H-representation of a joint space polytope that approximates, at any instant, the maximum range of joint displacements with respect to the system’s constraints is obtained in the form:

$$A_{k}\delta q_{k}\leq b_{k}.$$ (21)

The polytope can then be transformed to a V-representation using the double description method (Fukuda and Prodon, 1996), after which the Cartesian representation is obtained using the differential kinematic model (Eq. 2). For a full derivation of the above procedure, the reader is invited to refer to Long et al. (2019).

**FIGURE 2:**
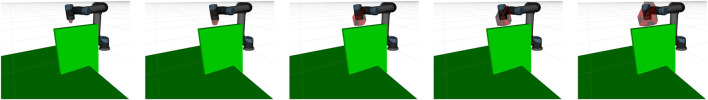
Visualization of the shrinking constrained motion polytope, with origin at robot tool frame. From left to right, the linearization limit is selected as *ϵ*_*lin*_ = 0.038, *ϵ*_*lin*_ = 0.060, *ϵ*_*lin*_ = 0.1, *ϵ*_*lin*_ = 0.117, *ϵ*_*lin*_ = 0.138. The decreasing linearization value shrinks the volume of free space within which the operator can move the end effector, thus guiding the manipulator towards a pre-defined goal configuration.

The derived *constrained motion polytope* bounds a volume within which the robot can move without violating any constraints. In the shared control application, it is also desirable to guide the user towards a goal configuration, hence we propose to use the polytope as a supplementary guide by modifying the extents based on proximity to a goal. In order to do so, the linearization limit, *ϵ*_*lin*_, is modified as a function of distance from the current end effector pose to the goal pose, as given in (Eq. 3). Consequently, the free space within which the robot can move is reduced. An example of this effect is shown in Figure 2.

During the teleoperation, the user’s input generates a desired twist ***ν***_*d*_ for the robot end effector, which is then converted into a joint velocity command *δ* **q**_*i*_ and sent to the robot controller by solving the following optimization problem using SNOPT (Gill et al., 2005):

$$\begin{array}{ll} & \underset{\delta q_{i}}{\text{minimize}}\|\nu_{d}-J_{i}\delta q_{i}\|\\ & \text{subject to }A_{i}\delta q_{i}\leq b_{i}. \end{array}$$ (22)

**q**_*i*_, **J**_*i*_, **A**_*i*_ and **b**_*i*_ denote the robot’s current joint configuration, kinematic Jacobian matrix and constrained motion polytope hyperplanes.

Equation 22 minimizes the error between the user input and the robot’s motion, however, the linear inequalities may reject feasible solutions that lie outside the linearization limits. To overcome this, a set of joint configurations are randomly sampled in the neighborhood of **q**_*i*_. Let **q**_*j*_ denote one of the random joint configurations, (Eq. 22) is then reformulated as follows:

$$\begin{array}{ll} & \underset{\delta q_{j}}{\text{minimize}}\left||\nu_{d}-J_{i}\left(\delta q_{j}+\left(q_{j}-q_{i}\right)\right)|\right|\\ & \text{subject to }A_{j}\delta q_{j}\leq b_{j}. \end{array}$$ (23)

where the controller input is re-defined as $\left(\delta q_{j}+\left(q_{j}-q_{i}\right)\right)$. This is repeated for each sampled joint state, such that the controller input that best realizes the desired user twist is obtained. The sampling method enables the user to move the robot freely between polytopes while respecting intrinsic and extrinsic constraints. Additionally, the samples enlarge the solution space for each user input albeit at the cost of computation time. Therefore, the constrained motion polytopes can be seen as an arbitration of user inputs to ensure that the resulting robot motions are collision free and respect joint position limits.

During task execution, if the intention estimation is sufficiently confident in the inferred goal from (Eq. 7), *i.e.,* the posterior probability is greater than some pre-defined confidence threshold, then the system begins to guide the user towards the goal configuration by progressively limiting user motion:

$$\epsilon_{lin}=\left\{\begin{array}{cc} \epsilon_{max},\; & if\;\epsilon_{lin}>\epsilon_{max}.\\ \epsilon_{min},\; & if\;\epsilon_{lin}<\epsilon_{min}.\\ w_{1}\cdot min\left(o_{t}\right),\; & \text{otherwise}. \end{array}\right.$$ (24)

where *ϵ*_max_ and *ϵ*_min_ are respectively the maximum linearization value beyond which the error associated with motion linearization is unacceptable and the minimum value beyond which the end effector is immovable, while *w*_1_ is a weight that scales the shrinking of the polytope.

Thus, the hyperplane constraints defined in (Eq. 21) gradually become more restrictive as the user intention becomes more apparent. This in turn means that the solutions obtained from (Eq. 23) are more heavily arbitrated for the precise motion required to complete the screwdriver task.

## 3 Virtual Reality System

The last component of our framework integral to remote teleoperation is the interface, for which we utilize the medium of VR. In remote robot teleoperation, VR has become more widely used in recent years and has been shown to exhibit higher preference and usability over traditional interfaces (Wonsick and Padir, 2020). VR also provides an opportunity to create more immersive and intuitive interfaces due to its capability of interacting and visualizing in 3D. To develop our proposed VR interface, we first introduce a system architecture in Section 3.1 that can teleoperate the robot manipulator model of Section 2.1 in the target screwdriver setting. Next, we present in Section 3.2 a variety of VR prototypes for teleoperation, as well as a pilot user study to gauge their efficacy. Finally, Section 3.3 outlines the VR interface derived from this initial study.

### 3.1 Architecture

**FIGURE 3:**
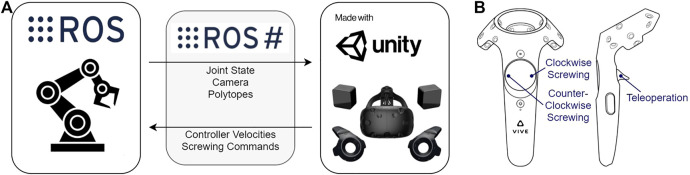
Diagram of the HTC Vive virtual reality system and handheld controller interface. **(A)** The entire virtual reality architecture is built along a ROS# communication bridge that transfers data between the robot (*e.g.,* joint state information) and Unity application (*e.g.,* command velocities). **(B)** Handheld controller with button functions to engage in teleoperation and screwing.

The overall architecture for the VR system is demonstrated in Figure 3A. In terms of hardware, the VR system is composed of an HTC Vive headset and a single controller. As for software, the VR application is developed using the Unity engine on the Windows operating system, while the robot is ROS (Quigley et al., 2009) compatible and runs on the Linux operating system. Therefore, to facilitate communication between VR and the robot, we employ an open-source software library called ROS#^1^. Rather than using position control, where waypoints are provided to navigate the arm, or trajectory control, where a generated path dictates the robot’s motion (Hetrick et al., 2020), we use a velocity controller to teleoperate the robot.

To teleoperate the arm through VR, we translate relative velocities originating from the Vive controller into command velocities for the robot’s end effector. Operators are able to activate velocity control by holding down a button assigned for teleoperation, as depicted in Figure 3B, and then move the controller in full 6 degrees-of-freedom. Teleoperation is automatically stopped whenever the button is released. Additionally, to assist in the screwing task, operators are provided with a means of rotating only the screwdriver, similar to a power drill. Figure 3B shows the buttons designated to rotate the screwdriver in either a clockwise or counterclockwise direction at a fixed velocity. Similar to the velocity control, rotations are only activated while the button is being held down and stopped upon release.

### 3.2 User-Centered Design

**FIGURE 4:**
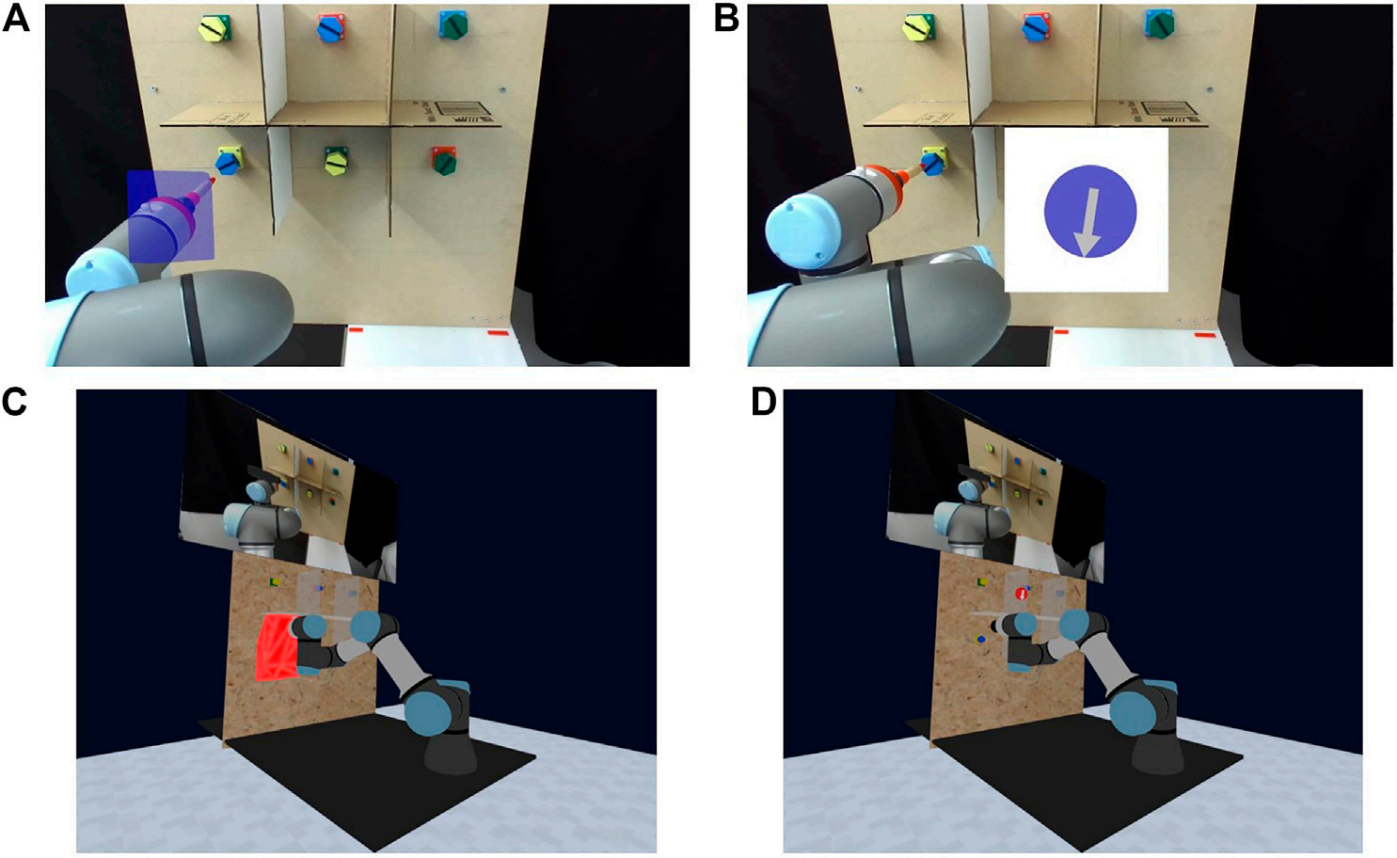
Four prototypes of visualizations for the virtual reality interface. **(A)** Camera + Polytope **(B)** Camera + Compass **(C)** Model + Polytope **(D)** Model + Compass.

Recent work involving shared control and mixed reality headsets, like the HTC Vive or Microsoft HoloLens, has stipulated that an interface must follow careful design considerations to avoid hindering task performance (Zolotas et al., 2018). Whilst prior studies have illustrated the flaws or benefits of different graphical cues when signaling robot intent during human-robot interaction (Walker et al., 2018; Zolotas and Demiris, 2019), each individual application will warrant a novel perspective. Consequently, we advocate following a user-centered design where human subjects helped resolve the final VR interface for our shared control system, which is presented in Section 3.3. This section describes four VR interface prototypes developed for the target setting of teleoperating a screwdriver with a robotic arm (portrayed in Figure 4), as well as the results from a pilot user study to evaluate these prototypes. These prototypes differ in two aspects: the environmental view and the choice of visual guide on the internal shared control.

Regarding the environment perspective, participants were shown interfaces that displayed either a pure camera view, Figures 4A,B, or a constructed model view of the relative elements of the robot’s environment, coupled with a smaller version of the former camera view, Figures 4C,D. For all four interface prototypes, a stereo camera was used to capture a 2.5D view of the environment. This camera was mounted behind the robot to provide a complete visual of the scene and robot itself.

As for visual guides, participants were shown either the direct polytope generated from Section 2.3, Figures 4A,C, or a circular disk with an arrow, *i.e.,* a *compass*, which incorporates information on the internal shared control in a reduced form, Figures 4B,D. In the compass visualization, there are three varying elements based on the internal shared control: the size of the circular disk, the direction of the arrow, and the color coding. The disk behaves and varies like the linearization limit from (Eq. 24) by only appearing once a goal estimate exceeds the aforementioned confidence threshold and scaling in size according to the end effector’s distance from the inferred goal. Meanwhile, the compass arrow is updated to always point to the center of the operator’s desired goal location based on the end effector’s current pose. Furthermore, both the polytope and compass visualizations are color-coded to indicate safety from obstacles. Using the ratio of reduction in space between the constrained and allowable motion polytope volumes, we color the compass red for constrained space and blue for wide allowable space. In other words, a smaller ratio highlights close proximity to obstacles with red to warn for danger, while blue signifies a larger ratio and a safer space for operation.

To assess the utility of these interfaces with respect to the screwdriver teleoperation task, we gathered 14 engineering students (1 female, 13 male) aged 19–29 (median: 25) to rate their preferences after viewing all the designs and provide general feedback of their impressions. It is worth noting that the entire subject pool reported familiarity with robotics, coinciding with the target user population of most teleoperation applications. Each participant was also presented a random sequence of the visualizations to offset any effects from ordering.

**FIGURE 5:**
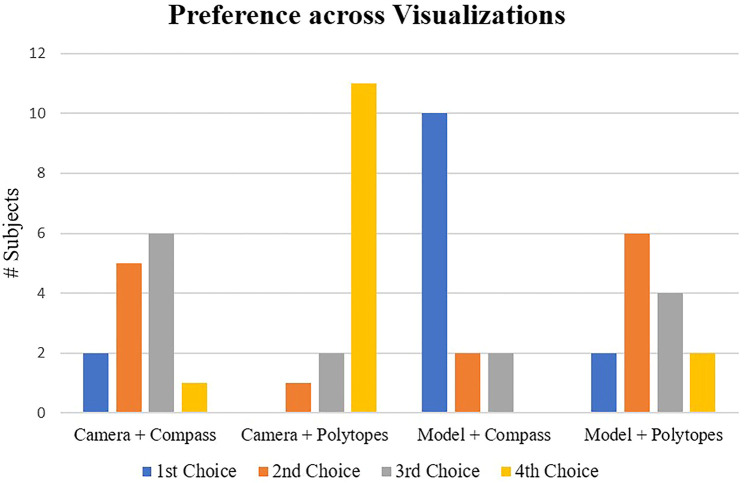
User preference ratings across the four visualization prototypes from the pilot study. There is a clear majority of “1st Choice” and “4th Choice” selections for the “Model + Compass” and “Camera + Polytopes” visualizations, respectively.

Participant ratings from this survey are illustrated in Figure 5, which demonstrate that the most and least preferred options are predominantly the “Model + Compass” and “Camera + Polytopes” configurations, respectively. There were a few remarks made by volunteers that are worth mentioning here. To begin with, several participants stated that the direct polytope visualization was not immediately intuitive and “hard to understand” or “confusing” as a visual guide. However, many highlighted that the concept of the polytope and how it encapsulates the robot’s internal state and environment could be useful if presented in alternative ways. Some participants also commented on how more time spent getting accustomed to their properties, or more “coaching”, may have improved their ratings. Another recurring statement was about having a zoomed in perspective of the bolts, as the stereo camera did not provide enough fidelity or depth perception for the task. Several participants selected the “Model” view as their preferred visualization because it helped them acquire perspective of the task and allowed them additional viewpoints. Other feedback points were to change the scaling of the compass size or increase the transparency of the polytope shading.

### 3.3 Final Interface

**FIGURE 6:**
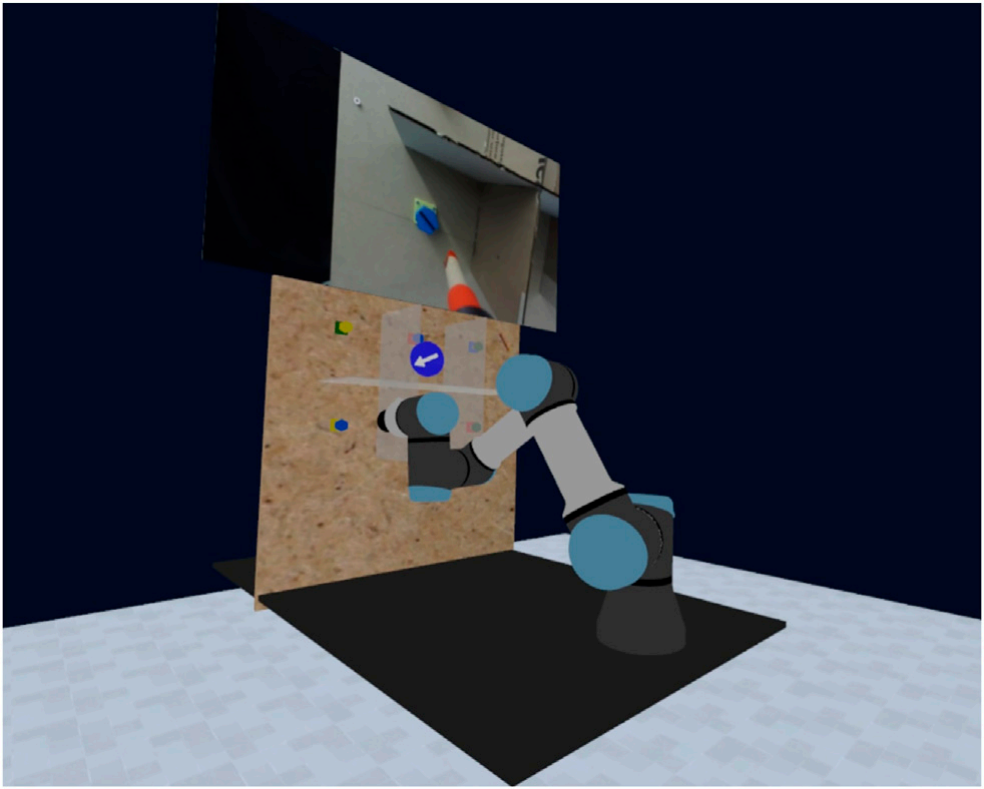
Final virtual reality interface bears similarity with the most popular “Model + Compass” prototype, except for two adjustments. First, the image displayed above the model now portrays the view captured from a mounted end effector camera. Second, the compass scaling in size has been adjusted according to user recommendations.

With the results from our user-centered design survey, we opted to use the model-based interface with the compass guide as our final interface. Based on the verbal feedback received during the study, we implemented two modifications to this interface. First, we moved the camera’s mount point from above-and-behind the robot, which gave a third-person point of view, to a first-person point of view where the camera was directly mounted to the end effector of the robot arm. This modification provided a closer look at the screwdriver and bolts. Second, we adjusted the minimum and maximum scale of the compass to help emphasize the distance to goal. The resulting interface is displayed in Figure 6. Despite tailoring this interface to the screwdriver teleoperation task, it could easily generalize to other applications that require both gross and fine manipulation.

## 4 Experiment

To evaluate our proposed VR system for shared control using motion polytopes, we conducted an experiment where subjects teleoperated a robotic arm to screw bolts tight. The chosen platform for this task was a UR3e collaborative robotic arm with a 3D printed extension at its end effector to hold a wooden screwdriver (refer to Figure 1). All other hardware related to the VR setup is described in Section 3.1. The shared control processes of intention estimation and arbitration via polytope constraints are implemented atop of the ROS (Quigley et al., 2009).

### 4.1 Experimental Setup and Protocol

A total of 14 volunteers (3 female, 11 male) aged 20–31 (median: 25) were recruited for the experiment. Unlike the pilot study to determine a VR interface, not all subjects had a robotics background for this experiment, thereby reflecting a broader community of potential operators for teleoperation activities. Indeed, three reported no robotics familiarity and there was an even split in those knowledgeable about VR. The purpose of this experiment was to assess whether different control modes and visualizations of the internal robot decision-making would impact an operator’s performance and overall experience when guiding a screwdriver. Three modes of control were examined: direct teleoperation (Mode “A”), shared control using joint space polytopes as described in Section 2 (Mode “B”), and shared control combined with the compass visualization from Section 3.3 (“B + Viz”).

The experimental setup and protocol are as follows. Subjects were initially provided with an information sheet outlining the task specifications before signing a consent form. To reduce the fatigue and workload incurred on participants, the task was simplified to only require a half-turn tightening of the two blue bolts on the wooden board (see bottom left of Figure 1). The ordering of different control modes was also randomized to counterbalance the effects of trial order. Prior to each trial, an optional training period with the handheld controller was offered to subjects, where they could operate the robotic arm without wearing the HTC Vive headset. Likewise, the VR interface and visualizations (*e.g.,* the compass) were explained to prevent any confusion on their purpose mid-trial. Moreover, subjects were instructed to perform the task as quickly and safely as possible, which coincides with our metrics for evaluation in the next section.

### 4.2 Evaluation Metrics

An array of quantitative and qualitative metrics is considered for this teleoperation experiment. We adopt two traditional quantitative metrics in the evaluation of shared control against direct teleoperation: time-to-completion and number of collisions. Both of these metrics are recorded manually by the experimenters, with collisions separated into two categories: major and minor. Minor collisions refer to instances where the robotic arm comes into any contact with obstacles, while major events are whenever the UR3e triggers an emergency stop, *e.g.,* due to excess force supplied at the end effector. In the case of emergency stops, a subject’s times would be discarded, and they would then proceed onto the next control mode. Our last quantitative measure is the mean temporal distance from environmental object constraints along the task, which can be regarded as an indicator of safety.

Nevertheless, these quantitative metrics do not account for human factors, such as user preference or cognitive workload. As a result, we opt to use two prevalent questionnaires within the field of human-robot interaction: the NASA-TLX (Hart and Staveland, 1988) and the System Usability Scale (SUS) created by Brooke (1996). Both rating scales have been applied in similar mixed reality studies (Brooks and Szafir, 2020; Rosen et al., 2020), with the NASA-TLX denoting perceived workload and the SUS relating to usability. Participants completed these questionnaires immediately after every trial. In addition to the NASA-TLX and SUS, users were also asked after their last experiment trial to provide general feedback via a survey. This feedback included answering three questions on a 5-point Likert scale to assert how *distracting*, *effective* and *predictable* the VR visualizations were at elucidating the shared control. At the end of the experiment, participants had to indicate their preferred mode of control.

### 4.3 Quantitative Results

**FIGURE 7:**
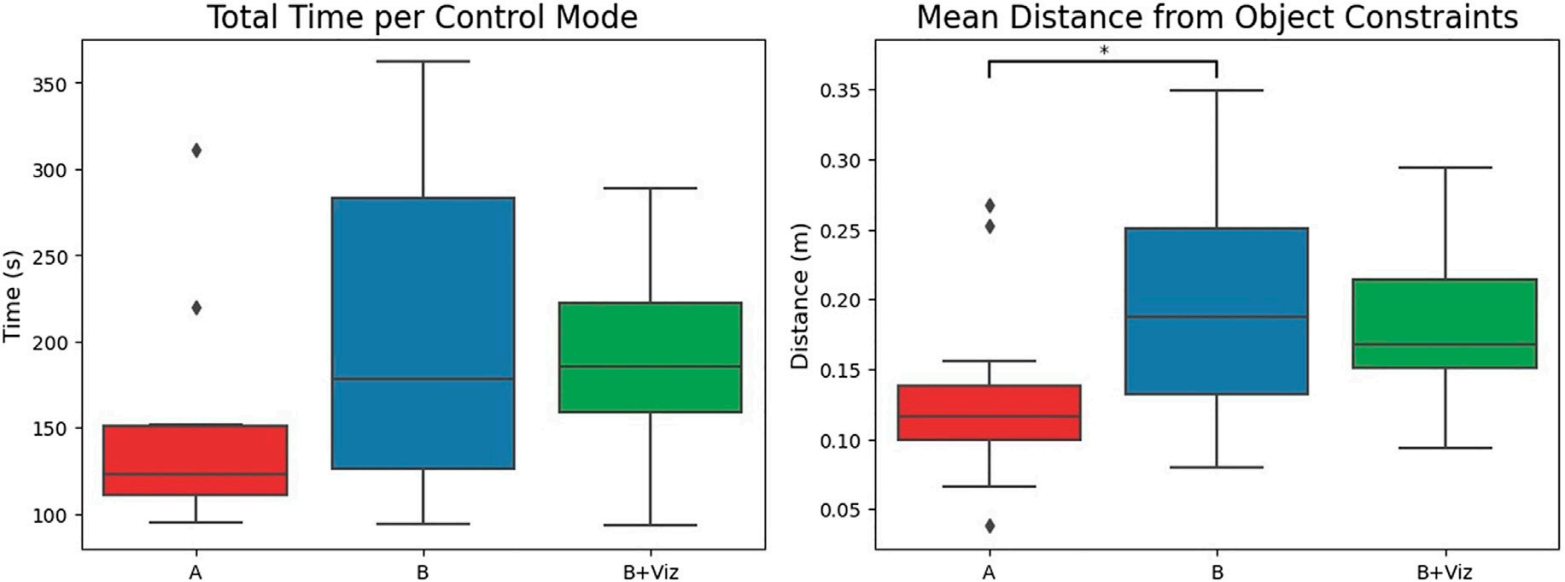
Total time taken and average distance from environmental obstacles when completing the screwdriver task using three different control modes. Direct teleoperation garners quicker times than the shared control modes (“B” and “B + Viz”) at the expense of safety, as signified by the closer proximity of trajectories to object constraints.

The quantitative results on total time taken are illustrated in the left-hand plot of Figure 7. A one-way repeated ANOVA signals a statistical effect on the basis of timing (*F* (2, 26) = 5.333, *p* = 0.011), yet a post-hoc analysis with Tukey’s HSD test finds no significance between direct teleoperation and shared control assistance (*p* > 0.05 for multiple comparisons). Though the general trend suggests quicker times when completing the task with mode “A” (148.1 ± 64.3s) than modes “B” (205.4 ± 95.4s) and “B + Viz” (192.5 ± 57.3s). This outcome might have been anticipated given that the shared controller is designed to constrain end effector motion in order to ensure safety.

To observe differences in safety, we refer to the average distance from obstacle constraints shown on the right of Figure 7. Running a one-way repeated ANOVA yields a statistical difference for this metric (*F* (2, 26) = 5.116, *p* = 0.013). Post-hoc analysis with Tukey’s HSD corrections indicates that joint state trajectories under direct teleoperation are at significantly closer proximity than those for shared control (*p* = 0.029), but not the visualization extension (*p* = 0.109). The lack of significance for mode “B + Viz” is possibly linked to the compass aid interfering with a user’s environmental perception. Regardless, it is clear that direct teleoperation possessed higher risk of contact with objects in the environment.

**FIGURE 8:**
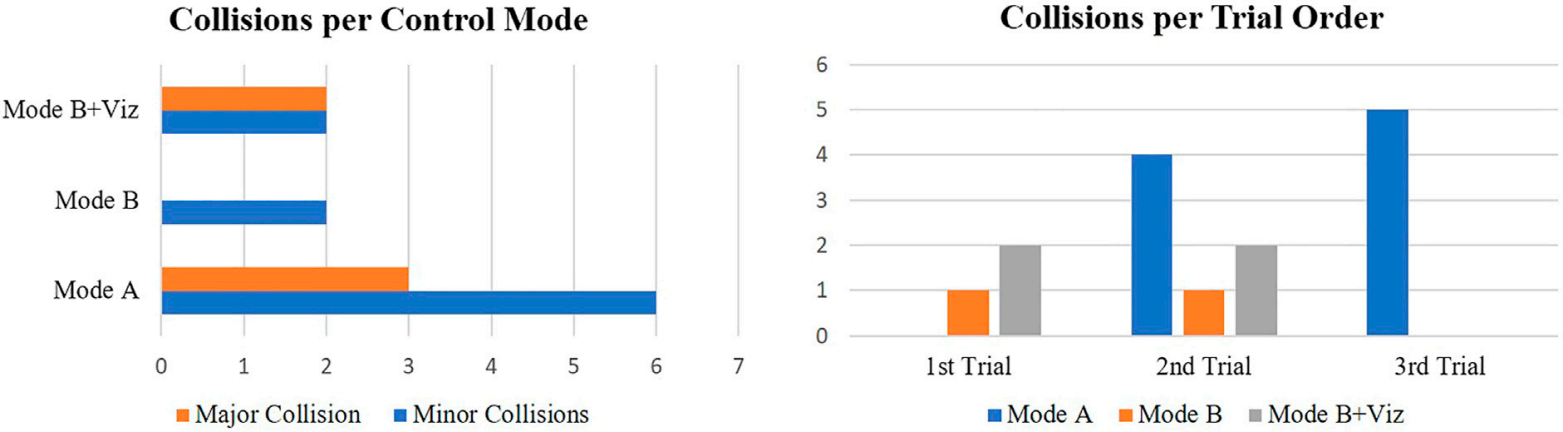
Number of collisions for the three control modes illustrated as a function of collision type and trial order. Direct teleoperation had a much higher collision frequency over shared control modes (“B” and “B + Viz”), despite only occupying a single run per subject. Any potential improvements derived from users learning the task across multiple trials also had no positive impact on the collision count for mode “A”.

The collision count in Figure 8 further reinforces this statement by demonstrating a higher frequency of both minor and major events under direct teleoperation. Given the nature of the task and how forceful contact was necessary to screw in the bolts, even “B” modes accrued some collisions. This is primarily because the bolts themselves could not be modelled as obstacle constraints for the motion polytopes. Another noteworthy observation about collisions is related to the effect of trial order (right-hand side of Figure 8). For shared control modes, collisions only occurred during the earlier trials where subjects were often still becoming accustomed to the task (emergency stops for “B + Viz” only happened on *first* trials). Whereas for direct teleoperation, collisions cluster around the latter trials with most of them accumulated on the *last* trial.

### 4.4 Survey Results

**FIGURE 9:**
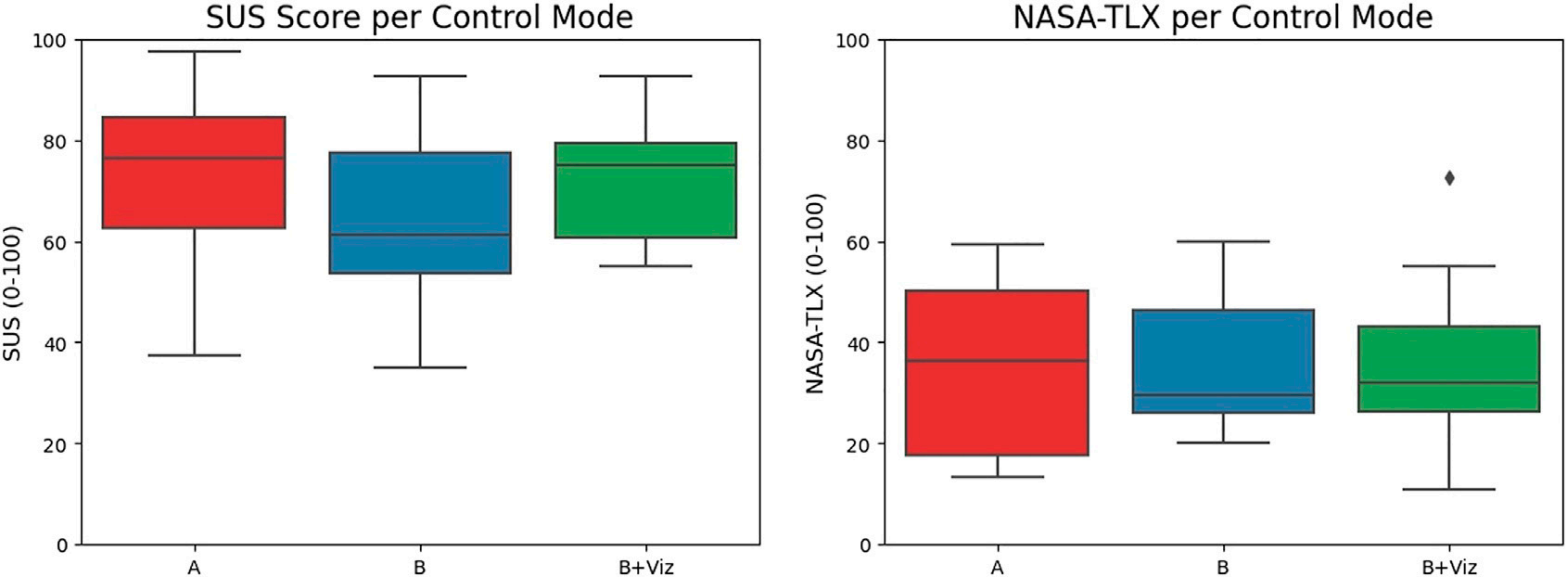
SUS score and NASA-TLX for the screwdriver task using the three different control modes. While the SUS score for shared control without visual feedback (mode “B”) are the lowest rating of usability, there are no significant differences between the three in perceived workload.

The survey responses to the SUS and NASA-TLX are summarized in Figure 9. A repeated ANOVA test reveals no main significant effects across the groups (*F* (2, 26) = 2.804, *p* = 0.079 for SUS; *F* (2, 39) = 0.101, *p* = 0.904 for NASA-TLX). Hence, there are no strong conclusions to be drawn from these qualitative results, except that the SUS score for mode “B” (62.3 ± 16.9) is marginally less than that of “A” (72.3 ± 16.9) and “B + Viz” (72.5 ± 12.3). These trends in scores suggest greater usability when either employing the reactive nature of direct teleoperation or the visual guidance of the compass in shared control. Also note that both mode “A” and “B + Viz” exceed an average usability score of 68 (Sauro, 2011), which is a positive outcome for this otherwise challenging screwdriver teleoperation task. As for the NASA-TLX, the perceived workload exerted by participants bears significant similarities across the control modes.

Table 1 contains the general perceptions of users on the VR interface. Subjects tended to rate the visualizations as *effective* and unlikely to cause *distraction*, but the lack of *predictability* for collisions hints at an ongoing model mismatch. More specifically, the color-coding of the compass was ineffective at warning participants of any imminent danger due to nearby obstacles. The preferred modes shown in Table 2 also delineate that there is an even split between direct teleoperation and shared control, implying that improvements are warranted before superior preference for mode “B + Viz” is attainable.

**TABLE 1:** General ratings on the visualizations from 1–5 (1 = strongly agree, 5 = strongly disagree).

Question	Mean Rating ± Std. Dev
I felt *distracted* by the visualizations	3.79 ± 1.42
I found the visualizations *effective* for the task	2.21 ± 1.31
I could *predict* collisions from the visualizations	3.00 ± 1.57

**TABLE 2:** Preference scores for the three control modes.

Mode A	Mode B	Mode B + Viz
7	3	4

## 5 Discussion

There are a few key insights drawn from this screwdriver teleoperation experiment. First and foremost, we observe that the rationale behind our polytope-based shared control was not made abundantly transparent to users and thus persists the problem of model misalignment. This is evident from the user’s ratings in Table 1, whereby the color-coded compass did not help operators anticipate collisions. A further testament to this point on model mismatch is that the “less reactive” nature of mode “B” was not immediately obvious to various subjects. For our target screwdriver setting, this “slow” behavior was particularly notable, as the shrinking polytope volumes had the adverse effect of preventing users from easily departing a bolt once it had been screwed tight. Various subjects commented on this effect with statements like “it was difficult to pull back” or “it slowed me down”.

Constrained motion is a byproduct of our shared control assistance, however it also has the beneficial effect of increasing safety. For instance, the arbitration procedure described in Section 2.3 helped stabilize operator control when zoning in on bolts and thus reduced the number of collisions with the enclosing borders of the environment. Likewise, the mean distance maintained from obstacle constraints along shared control trials was significantly better than direct teleoperation, accounting for improved safety. While collisions persisted across all control modes, there were none recorded for users with shared control on their final trial. This pattern hints that subjects had either formed a dependency on the safe motion adjustments of the arbitration, or that any potential improvements procured from learning the task could not translate into performance gains with direct teleoperation.

Another finding from this study revolves around user preference. Even after improving the success rate of participants in shared control modes by reducing the number of failed attempts due to major collisions, user preference was still split across the modes. Although speed is not necessarily correlated with user preference and there are other factors at play, such as performance and transparency (Dragan and Srinivasa, 2013), it can still influence people’s opinions in toy experiment settings like ours. An example of this is when one subject opted for direct teleoperation as their favorite control setting after successfully completing the task with modes “B” and “B + Viz”, despite triggering an emergency stop on mode “A”. We stipulate that by simulating incentives to complete the task *successfully*, *e.g.,* offering money, user preferences may have swayed towards favoring shared control.

Lastly, it is worth mentioning that our setup to remote teleoperate a robot wielding a screwdriver is only preliminary and possesses certain limitations. For example, a prominent drawback of our study are its low subject numbers, meaning the results are unlikely to hold sufficient statistical relevance. Moreover, the screwdriver and bolts are from a children’s wooden tool set, which simplify the task but reduce it down to a toy setting. As mentioned earlier, the bolts are not modelled as constraints for the polytopes due to the force exertion necessary for screwing, and so the task is not fully accommodated by the shared control methodology. Regardless of these diminutions, the overall experiment strives to mimic real-world applications of remote teleoperation and we hope that it inspires further studies to adopt similar setups.

## 6 Future Work

A critical finding of our study is that the compass aid derived from the pilot survey was unable to successfully improve the predictability of collisions during teleoperation. In spite of its clear and non-distracting purpose, the shared control remained somewhat elusive to users. Based on this finding and user suggestions, we suspect that an important avenue for future research on model misalignment in shared control is to explore longer term human-robot interaction.

Recent teleoperation frameworks have addressed model mismatch through immersive interface design (Zolotas and Demiris, 2019; Brooks and Szafir, 2020), however the literature is sparse in studies that investigate the role of lifelong assistance (Demiris, 2009). In our pilot survey, numerous participants were appealed by the idea of directly visualizing polytopes, yet struggled to build an intuition for them in the short span of a quick trial. Another encouraging result of our teleoperation experiment was the lack of collisions in later trials under shared control, which prompts an examination into the learning curves of different users. We thus hypothesize that a study into more complex virtual guides for shared control, like polytopes as geometrical objects, require longer experiment scales, *e.g.,* trials spread out across multiple days or interactions. While no haptic interface was incorporated into our proposed framework, future architectures should also endeavor to issue multimodal feedback (Losey et al., 2018), especially for physical tasks like screwing bolts.

An additional course for inquiry will be to improve the means of generating polytopes during arbitration. At present, the constrained motion polytopes only account for robot and environmental constraints, but they could also be tailored to individual user preferences. A simple use-case of this notion could be to dynamically adjust the linearization limit from within the VR interface, thereby allowing the operator a personalized experience. Furthermore, it should be noted that the polytopes are generated at run time in response to robot configuration and environmental changes. At present, an environment model is loaded during the initialization and then added to a maintained collision world. This environment comprises a set of objects defined by collision models, either described by solid primitives or meshes, and pose information. Objects can be added or removed at run time, but this process is cumbersome and impractical. Ongoing work is focusing on integrating *octomaps* (Hornung et al., 2013), 3D occupancy grids based on real-time sensor data, to enable the generation of motion polytopes from dynamic point cloud data.

Finally, our algorithm does not currently respect real-time constraints. Cycle time is minimized by reducing the mesh density of collision objects, but in future work we believe real-time constraints can be satisfied by the following measures. First the control frequency can be further increased by isolating the polytopes from the control loop. In doing so, the polytopes can be generated or updated periodically, independent of user input commands, allowing teleoperation within a feasible space at a much faster cadence. Second, we used SNOPT (Gill et al., 2005) to solve (Eq. 22), but real-time performance could be obtained by using an anytime optimization solver (*e.g.,* an interior point method Wachter, 2002) that returns a feasible solution at any termination point.

## 7 Conclusion

In this paper, we introduced a shared control method for teleoperation using constrained motion polytopes and developed a corresponding VR interface. The novelty over previous virtual fixture methods for arbitration in shared control was the introduction of polytopes through the notion of “shrinking” linearization limits. To accommodate remote teleoperation, a model-based VR interface was also presented to guarantee effective telepresence. A pilot survey was first conducted to inform the design of this VR interface and help avoid misunderstandings about the underlying shared control. The resulting system was then evaluated in a human-robot interaction experiment involving a UR3e arm extended with a screwdriver, so as to discern its performance and promise for remote manipulation.

Our experimental results reinforce the usability of our proposed VR system for the screwdriver manipulation task. In particular, we observed that while slower than direct teleoperation, the shared control led to increased safety. Moreover, the shrinking volume of the polytopes prevented erratic motion at close proximity to the bolts, which in turn reduced the likelihood of failing the task. Despite the challenging nature of this screwdriver task, most participants successfully completed every trial using our VR interface, citing the experience as fun and enjoyable.

## Data Availability

The original contributions presented in the study are included in the article/Supplementary Material, further inquiries can be directed to the corresponding author.

## Footnotes

1
https://github.com/siemens/ros-sharp
